# Establishment of a novel obesity mouse model: the induction of intestinal microbiota dysbiosis

**DOI:** 10.1038/s41598-024-63964-2

**Published:** 2024-06-11

**Authors:** Qiuju Li, Xiaolin Gao, Ruizhen Jia, Jianjun Deng, Chaomin Wan

**Affiliations:** 1 Department of Paediatrics, West China Second University Hospital of Sichuan University, Number 20, 3rd Section, People’s South Road, Chengdu, 610041 Sichuan China; 2 Key Laboratory of Birth Defects and Related Diseases of Women and Children (West China Second University Hospital of Sichuan University), Ministry of Education, Chengdu, China; 3 Open Laboratory, West China Second University Hospital of Sichuan University, Chengdu, 610041 Sichuan China

**Keywords:** Obesity, Mouse model, Intestinal microbiota dysbiosis, Microbiology, Gastroenterology

## Abstract

To establish and evaluate an intestinal microbiota dysbiosis-induced obesity mouse model. 50 C57BL/6 J male healthy mice were randomly divided into an obesity model group and the control group. The body weight, body length, and Lee’s index of the two groups of mice at week 1 and week 10 were compared. Serum glucose (GLU), total cholesterol (TC) and triglyceride (TG) were measured by enzyme-labeled colorimetric methods. Illumina HiSeq 16S rDNA high-throughput sequencing technology was used to characterize intestinal microbiota in feces. The success rate of model establishment in obese mice was 52%. The body weight, body length, Lee’s index, and abdominal fat (wet weight) in the obese model group were all higher than those in the control group, and the differences were statistically significant (*P* < 0.01). Serum GLU and TC levels in the obesity model group were higher than those in the control group (*P* < 0.05), and there was no difference in TG levels between the two groups (*P* > 0.05). The control group contained more abundant intestinal microbiota phyla and genera than did the obesity model group; the differences between the two groups were significant (*FDR* ≤ 0.05, *P* ≤ 0.05). Intestinal microbiota dysbiosis can be used to generate an obesity model in mice.

## Introduction

Obesity is a systemic disease that causes the excessive accumulation of adipose tissue. It is caused by various factors and has an extensive impact on metabolism and the endocrine, cardiovascular, and other organ systems. It is also an important causative factor of diabetes, hypertension, cardiovascular diseases and other major diseases^1,2^. As a recognized major public health problem, obesity has received increasing attention from researchers in China and abroad^1–3^. In recent years, studies have shown that the intestinal microbiota plays an important role in the development and regulation of obesity^4,5^. Researchers in China and around the world have used high-fat and high-sugar methods to establish mouse models of obesity and have successfully carried out a number of studies on this basis^5–7^. However, there have been few studies on mouse models of obesity induced by intestinal microbiota dysbiosis. We used ampicillin, neomycin, metronidazole, and vancomycin to establish an intestinal microbiota dysbiosis-induced obesity model. We analysed and compared the differences in physical characteristics, serum glucose (GLU), total cholesterol (TC), and triglyceride (TG) levels, and faecal intestinal microbiota characteristics between the model group and the control group. Moreover, we evaluated the modelling method to provide a basic experimental basis for further research on the mechanism, prevention, and treatment of obesity.

## Materials and methods

### Statement

All methods were carried out in accordance with relevant guidelines and regulations and were reported in accordance with the ARRIVE guidelines. This study was approved by the Ethical Committee on Animal Experimentation of the Ethics Committee of West China Second Hospital Sichuan University (No. 2020008).

### Animals

Healthy SPF-grade C57BL/6 J mice (male; 6 weeks old; body weight, 23–28 g) and their diet were purchased from Chengdu Quan Xi Biotechnology Co., Ltd. The mice were adaptively fed for 1 week before the experiment. Five mice were housed per cage. The humidity was controlled within a range of 50 to 70%, and the temperature was maintained within a range of 20 to 24 °C, with a 12-h light/dark cycle. The animals had ad libitum access to food and water. The water consumption per mouse was 4–5 ml/d. There were no differences in feed intake between the groups.

### Experimental design

The mice were randomly divided into 2 groups by SPSS 19.0 software, namely, the obesity model group and the control group, with 25 mice in each group. Mice in the obesity model group received continuous intragastric gavage of ampicillin, neomycin, metronidazole, and vancomycin for 7 days (10 mg of each antibiotic per day + 1 ml of sterilized water per mouse, for a total of approximately 1.1 ml, once per day). The above antibiotics (ampicillin, neomycin and metronidazole (1 g/L each)) and vancomycin (500 mg/L) were then added to the drinking water for the entire observation period^8–10^. Mice in the control group received continuous intragastric gavage of 1 ml sterilized water per day for the same period of time (1 time per day for 7 days). Both groups were fed an SPF diet (according to the Oriental Test).

The feeding conditions and living environment of the two groups were identical, and the experimental observation period was 10 weeks. Two grams of fresh faeces was collected at fixed time points (week 1 and week 10) and stored in an ultralow temperature freezer. All mice were euthanized by pentobarbital at week 10. Abdominal white adipose tissue was collected for examination. Blood samples were collected, centrifuged, and stored in a − 20 °C freezer until analysis.

### Measurements

Body weight and body length were measured, and Lee’s index was calculated for the two groups at week 1 and week 10. Illumina HiSeq16S rDNA high-throughput sequencing technology was used to compare faecal DNA from the two groups of mice (Oebiotec Co., Ltd.), and bioinformatics software was used to characterize the intestinal microbiota. Serum GLU, TC, and TG were measured by a test kit (ELASA, by Treasure Biological Company), and abdominal fat (wet weight, g) was compared between the two groups.

### Statistical analysis

SPSS 19.0 was used for statistical analysis of the data. Normally distributed data are expressed as the mean ± standard deviation ($\overline{\chi }$ ± s). Differences between the two groups were analysed using two independent sample *t* tests, and *P* < 0.05 indicated that a difference was statistically significant.

### Bioinformatics analysis

Illumina HiSeq 16S rDNA High-Throughput Sequencing Technology 2.0 was used to determine the V3-V4 region sequence of the microorganisms. The data were subjected to species classification, abundance analysis, and diversity analysis using Heatmap, PCA, Cluster, and Metastates software.

## Results

### Comparison of basic physiological indicators

There was no statistically significant difference in body weight, body length, or Lee’s index between the two groups of mice in the first week (*P* > 0.05). Nearly 50% of the mice in the obesity model group showed mild diarrhoea (untreated diarrhoea) and less movement at week 3, but these symptoms spontaneously improved at week 5. The body weights and body lengths of the mice in the obesity model group were greater than those of the mice in the control group beginning at week 7 (*P* < 0.05). At the end of modelling, i.e., at week 10, the body weight, body length, Lee’s index and abdominal fat (wet weight) of the mice in the obese model group were greater than those of the mice in the control group (*P* < 0.05) (Table 1). Among them, 13 mice weighed 20% above the average weight of mice in the control group^11,12^, with a modelling rate of approximately 52%. No mice died during the entire modelling period.

**Table 1:** Comparison of basic physiological indicators (10th week) ($\overline{\chi }$ ± s, *n* = 25).

	Body weight (g)	Body length (cm)	Lee’s index	Abdominal fat (wet weight) (g)
The obese model group	32.60 ± 5.44	9.84 ± 0.20	323.6 ± 13.21	4.75 ± 0.42
The control group	26.75 ± 1.70	9.38 ± 0.12	318.54 ± 0.86	3.91 ± 0.44
*t*	5.132	9.746	1.822	6.28
*P*	0.000	0.025	0.000	0.000

### Comparison of blood biochemical indicators

The differences in the serum GLU and TC levels between the obesity model group and the control group at the 10th week were significant (*P* < 0.05), but the differences in the TG levels were not statistically significant (*P* > 0.05) (Table 2).

**Table 2:** Comparison of blood biochemical indicators ($\overline{\chi }$ ± s, mmol/L, *n* = 25).

	GLU	TC	TG
The obese model group	6.89 ± 1.39	4.01 ± 1.28	1.17 ± 0.46
The control group	6.12 ± 1.07	2.93 ± 0.44	1.10 ± 0.51
*t*	2.19	3.94	0.48
*P*	0.03	0.01	0.06

### Bioinformatics analysis

**Figure 1:**
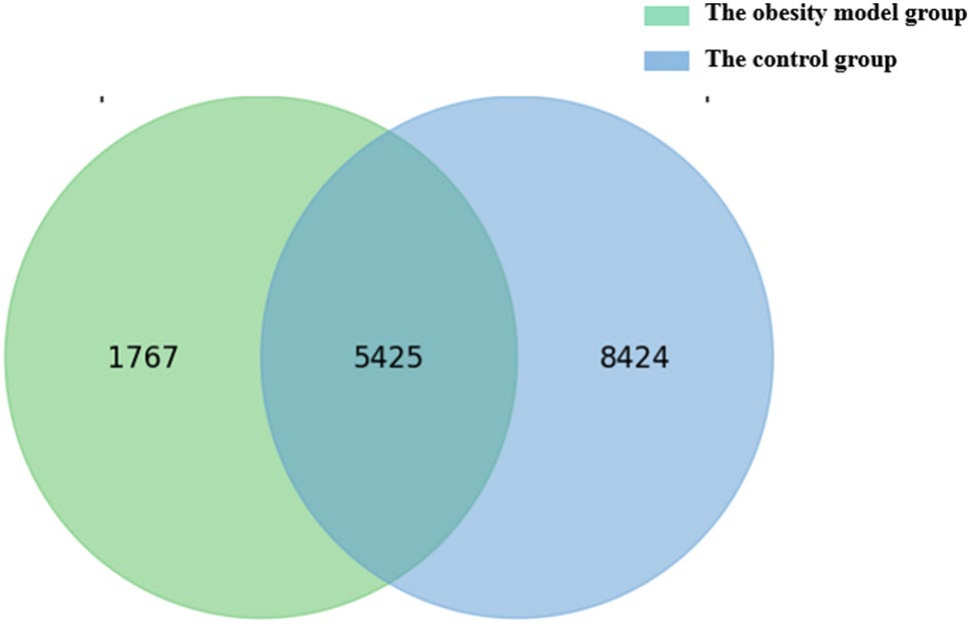
OTU venn figure.

At week 1, there was no difference in the DNA sequencing results of the faecal specimens from the two groups of mice. At week 10, faecal DNA obtained from the two groups of mice was sequenced using the Illumina HiSeq platform. A total of 15,616 valid 16S rDNA sequences were obtained after conducting splicing quality control with the software. There were a total of 5425 individuals in the obesity model group and control group; 1767 were specific to the obesity model group, and 8424 were specific to the control group, and the difference was statistically significant (*P* < 0.01) (Fig. 1).

### α-diversity analysis

**Figure 2:**
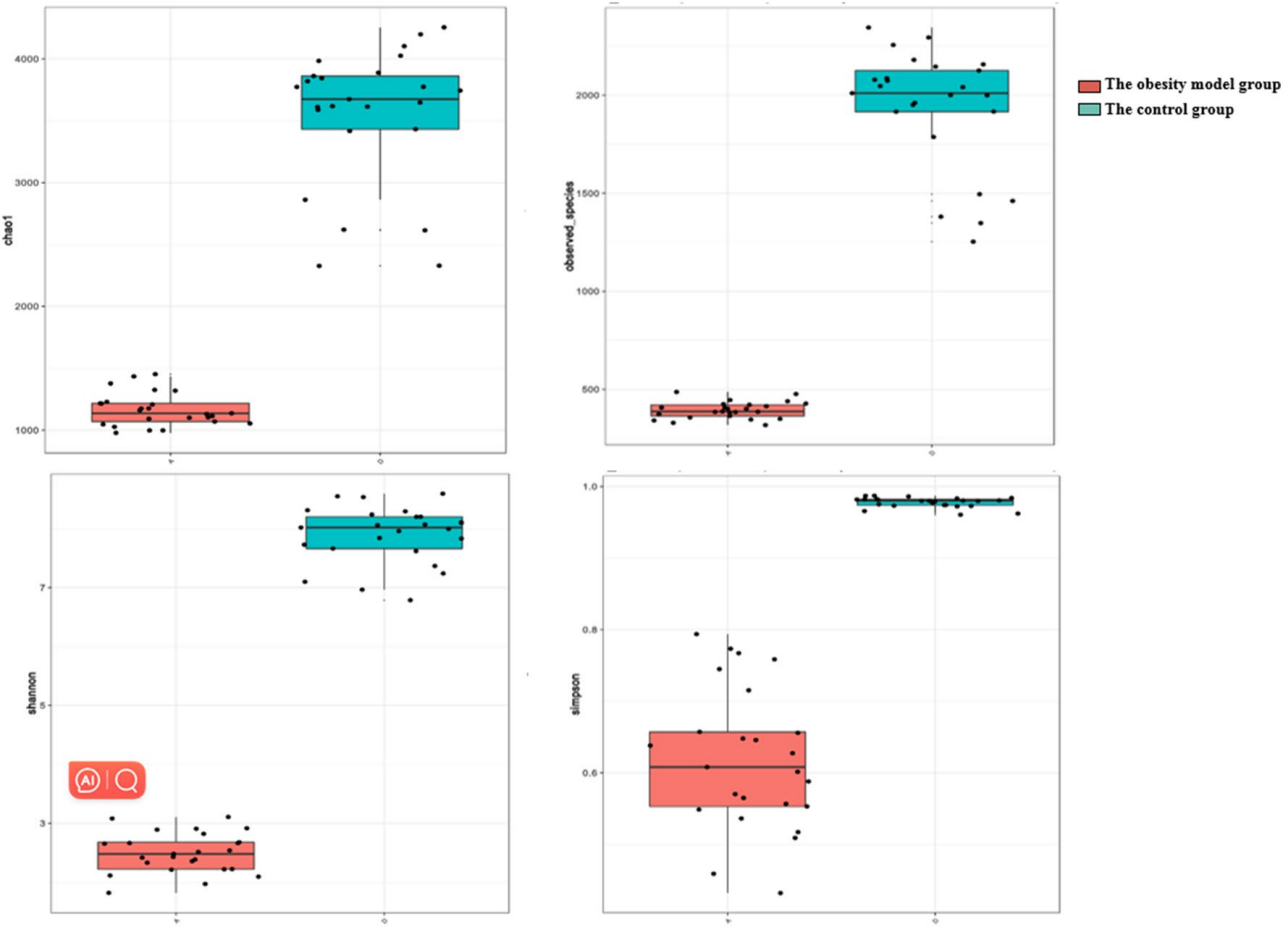
Box-plot of α diversity of the obesity group and the control group.

The Chao1 index, observed species index, Shannon index, and Simpson index values indicated that the difference in α diversity between the obesity model group and control group was statistically significant (*P* < 0.01) and that there were differences in species diversity (Fig. 2).

### β-diversity analysis

**Figure 3:**
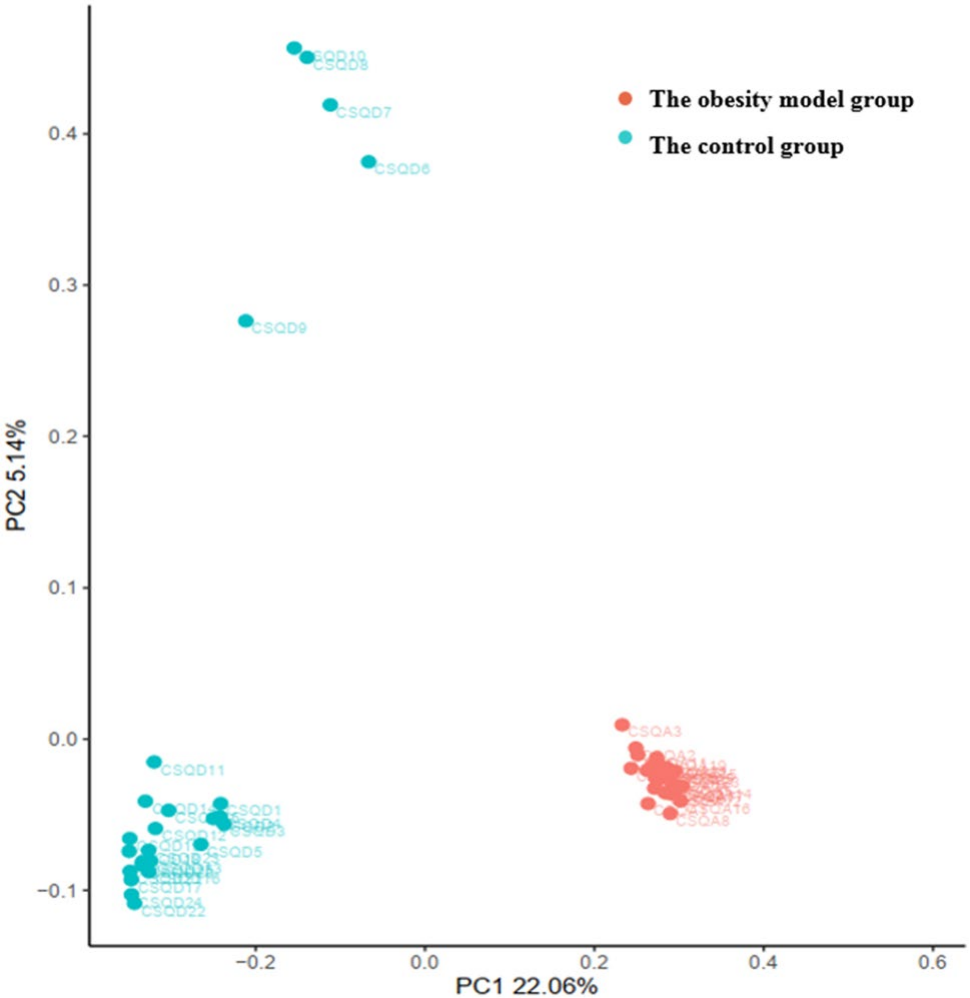
PCoA analysis of the obesity group and the control group based on OTU abundance.

Figure 3 shows the distribution of 50 samples (red dots = obesity model group, blue dots = control group). PC1 was the first principal coordinate, and the representativeness of the overall microbiota was 22.06%; the ordinate was PC2, and the representativeness of the overall microbiota was 5.14%. In the figure, the red points are located at the bottom right, and there was a clustering phenomenon. Most of the blue points were clustered at the bottom left, and only 5 points were scattered at the top left.

### Analysis of the abundance and differences in microbiota phyla and genera

**Figure 4:**
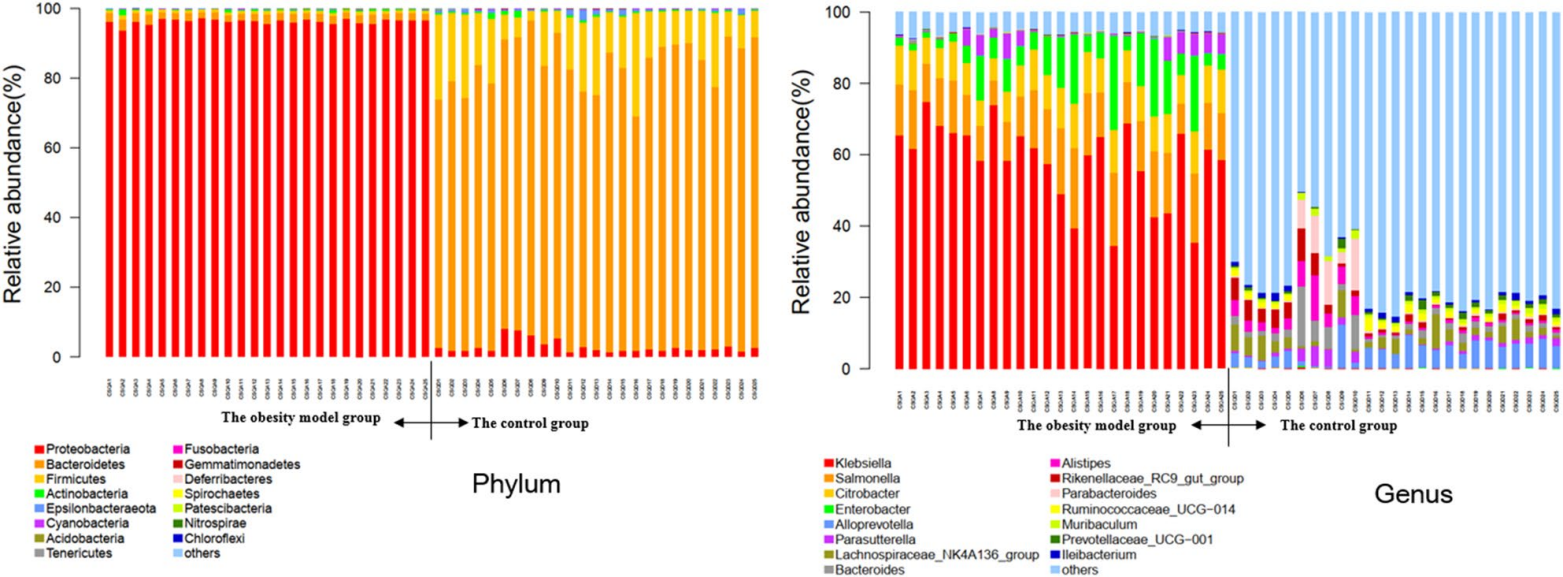
Histogram of the abundance distribution of phylum and genus of intestinal microbiota (Top15).

A total of 24 bacterial phyla were identified at the phylum classification level. The microbial phyla in the control group were more abundant than those in the obesity model group. Twelve phyla were significantly different between the two groups (*FDR* ≤ 0.05, *P* ≤ 0.05). The obesity model group was dominated by the *Proteobacteria* phylum, with 95.23% relative abundance, and the abundances of the *Fusobacteria*,* Patescibacteria* and *Spirochaetes* phyla were greater than those in the control group. The control group was dominated by the *Bacteroidetes* phylum, with a 76.82% relative abundance, and the abundances of *Firmicutes*,* Epsilonbacteraeota*,* Tenericutes*,* Gemmatimonadetes*,* Deferribacteres*,* Actinobacteria*, and other phyla were greater than those in the obesity model group (see Phylum in Fig. 4).

A total of 778 bacterial genera were detected at the genus level. The control group had a greater abundance of microbiota genera than did the obesity model group. A total of 119 genera were significantly different between the two groups (*FDR* ≤ 0.05,* P* ≤ 0.05). The obesity model group was dominated by the genus *Klebsiella*, with a relative abundance of 58.41%, and the abundances of the genera *Enterobacter*,* Salmonella* and *Citrobacter* were greater than those in the control group. The control group was dominated by unclassified genera, with 51.32% relative abundance, and the abundances of the genera *Alloprevotella*,* Lachnospiraceae*,* Bacteroides*,* Alistipes*,* Rikenellaceae* and *Parabacteroides* were greater than those in the obesity model group (Fig. 4).

## Discussion

With improvements in human living standards, the incidence of obesity has increased annually, resulting in varying degrees of harm to human health^3,13,14^. The pathogenesis of obesity is multifaceted and complex. At present, it is generally recognized that genetic and environmental factors are the main causes of obesity^3,16^. Recent studies have shown that the intestinal microbiota, an environmental factor, is closely related to the occurrence and development of obesity^4,5,15,16^. Ilseung et al.^18^ generated a model of adiposity by administering subtherapeutic antibiotic therapy to young mice and evaluated changes in the composition and capabilities of the gut microbiome. Laura et al.^19^ discovered that disruption of the microbiota during maturation by low-dose antibiotic exposure can alter host metabolism and adiposity. The relationship between the intestinal microbiota and the mechanism of action in obesity has become a research hotspot with regard to the prevention and treatment of obesity. Therefore, it is particularly important to establish an animal model of obesity caused by dysbiosis of the intestinal microbiota.

Our study revealed that the body weight, body length, Lee’s index, and wet weight of abdominal fat in the obesity model group were significantly greater than those in the control group (*P* < 0.01). Therefore, intestinal microbiota dysbiosis can induce obesity in mice. However, the modelling time was longer than that needed to induce obesity using a high-fat diet, and the success rate was low, i.e., only 52%. Forty-eight percent of the models were not successful in the obese model group because obesity may be related to the inflammatory response. We speculated that the use of antibiotics only plays a role in the inflammatory response to obesity and may not have an effect on other factors, such as diet, which warrants further research. Another reason we considered was that antibiotics increase feed efficiency in farm animals because they suppress the growth of bacteria that provide nutrients to their hosts. We suggest that this model can be used as a basis for the study of obesity caused by intestinal microbiota dysbiosis but not as a model for the study of conventional obesity. In this study, the serum GLU and TC levels in the obesity model group were greater than those in the control group (*P* < 0.05), but there was no difference in the TG levels between the two groups (*P* > 0.05). Most studies have reported dyslipidaemia in mouse models of obesity induced by a high-fat diet^20,21^. However, in this study, there was only an increase in body fat in the obesity model group, and there was no difference in blood TG levels compared with those in the control group. The specific mechanisms of action need to be further explored and studied. In this study, we used ampicillin, neomycin, metronidazole, and vancomycin for continuous intragastric gavage for 7 days, followed by inclusion of these same components in drinking water. The control group had greater abundances of intestinal microbiota phyla and genera than did the obesity model group. The differences in 12 bacterial phyla and 119 genera between the two groups were significant (*FDR* ≤ 0.05,* P* ≤ 0.05). The obesity model group was dominated by the *Proteobacteria* phylum and *Klebsiella* genus, and the control group was dominated by the *Bacteroidetes* phylum and unclassified genera. The use of antibiotics to generate intestinal microbiota dysbiosis was successful and can be used in future research to study the relationship between the intestinal microbiota and obesity. The mice exhibited mild diarrhoea and less movement during the 3rd week of the experiment, possibly due to side effects of the antibiotics. However, the above symptoms gradually improved spontaneously. It is possible that intestinal microbiota dysbiosis recovered without the use of large doses of antibiotics, as the drugs were only provided in small amounts in drinking water. The development of antibiotic resistance may also be an issue. In addition, whether the modelling of intestinal microbiota dysbiosis-induced obesity can be achieved faster, with higher modelling rates, needs to be explored in future studies.

## Conclusion

The intestinal microbiota dysbiosis-induced obesity mouse model generated in this study is simple and easy to establish. Moreover, the modelling conditions are quantifiable and controllable, and the model is basically stable. In the absence of a more complete study, this model can be used as the preliminary basis for the study of obesity caused by intestinal microbiota dysbiosis, providing a reference for additional studies of the mechanism, diagnosis and treatment of obesity.

## Data Availability

The datasets used and/or analyzed during the current study available from the corresponding author on reasonable request.

## Abbreviations

GLU: Serum glucose
TC: Total cholesterol
TG: Triglyceride
