# Author Correction: Establishment of a novel obesity mouse model: the induction of intestinal microbiota dysbiosis

**DOI:** 10.1038/s41598-024-67060-3

**Published:** 2024-07-10

**Authors:** Qiuju Li, Xiaolin Gao, Ruizhen Jia, Jianjun Deng, Chaomin Wan

**Affiliations:** 1 Department of Paediatrics, West China Second University Hospital of Sichuan University, Number 20, 3rd Section, People’s South Road, Chengdu, 610041 Sichuan China; 2 Key Laboratory of Birth Defects and Related Diseases of Women and Children, West China Second University Hospital of Sichuan University, Ministry of Education, Chengdu, China; 3 Open Laboratory, West China Second University Hospital of Sichuan University, Chengdu, 610041 Sichuan China

Correction to: *Scientific Reports* 10.1038/s41598-024-63964-2, published 11 June 2024

In the original version of this Article, Qiuju Li was incorrectly affiliated with ‘West China Clinical Medical College, Sichuan University, Chengdu, 610041, Sichuan, China’.

The correct Affiliation is listed below:

‘Department of Paediatrics, West China Second University Hospital of Sichuan University, Number 20, 3rd Section, People’s South Road, Chengdu 610041, Sichuan, China’.

The original Article has been corrected.

